# SHRiMP: Accurate Mapping of Short Color-space Reads

**DOI:** 10.1371/journal.pcbi.1000386

**Published:** 2009-05-22

**Authors:** Stephen M. Rumble, Phil Lacroute, Adrian V. Dalca, Marc Fiume, Arend Sidow, Michael Brudno

**Affiliations:** 1 Department of Computer Science, University of Toronto, Toronto, Ontario, Canada; 2 Department of Computer Science, Stanford University, Stanford, California, United States of America; 3 Department of Genetics, Stanford University, Stanford, California, United States of America; 4 Department of Pathology, Stanford University, Stanford, California, United States of America; 5 Banting & Best Department of Medical Research, University of Toronto, Toronto, Ontario, Canada; University of British Columbia, Canada

## Abstract

The development of Next Generation Sequencing technologies, capable of sequencing hundreds of millions of short reads (25–70 bp each) in a single run, is opening the door to population genomic studies of non-model species. In this paper we present SHRiMP - the SHort Read Mapping Package: a set of algorithms and methods to map short reads to a genome, even in the presence of a large amount of polymorphism. Our method is based upon a fast read mapping technique, separate thorough alignment methods for regular letter-space as well as AB SOLiD (color-space) reads, and a statistical model for false positive hits. We use SHRiMP to map reads from a newly sequenced *Ciona savignyi* individual to the reference genome. We demonstrate that SHRiMP can accurately map reads to this highly polymorphic genome, while confirming high heterozygosity of *C. savignyi* in this second individual. SHRiMP is freely available at http://compbio.cs.toronto.edu/shrimp.

## Author Summary

Next Generation Sequencing (NGS) technologies are revolutionizing the way biologists acquire and analyze genomic data. NGS machines, such as Illumina/Solexa and AB SOLiD, are able to sequence genomes more cheaply by 200-fold than previous methods. One of the main application areas of NGS technologies is the discovery of genomic variation within a given species. The first step in discovering this variation is the mapping of reads sequenced from a donor individual to a known (“reference”) genome. Differences between the reference and the reads are indicative either of polymorphisms, or of sequencing errors. Since the introduction of NGS technologies, many methods have been devised for mapping reads to reference genomes. However, these algorithms often sacrifice sensitivity for fast running time. While they are successful at mapping reads from organisms that exhibit low polymorphism rates, they do not perform well at mapping reads from highly polymorphic organisms. We present a novel read mapping method, SHRiMP, that can handle much greater amounts of polymorphism. Using *Ciona savignyi* as our target organism, we demonstrate that our method discovers significantly more variation than other methods. Additionally, we develop color-space extensions to classical alignment algorithms, allowing us to map color-space, or “dibase”, reads generated by AB SOLiD sequencers.

## Introduction

Next generation sequencing (NGS) technologies are revolutionizing the study of variation among individuals in a population. The ability of sequencing platforms such as AB SOLiD and Illumina (Solexa) to sequence one billion basepairs (gigabase) or more in a few days has enabled the cheap re-sequencing of human genomes, with the genomes of a Chinese individual [1], a Yoruban individual [2], and matching tumor and healthy samples from a female individual [3] sequenced in the last few months. These resequencing efforts have been enabled by the development of extremely efficient mapping tools, capable of aligning millions of short (25–70 bp) reads to the human genome [4]–[10]. In order to accelerate the computation, most of these methods allow for only a fixed number of mismatches (usually two or three) between the reference genome and the read, and usually do not allow for the matching of reads with insertion/deletion (indel) polymorphisms. These methods are extremely effective for mapping reads to the human genome, most of which has a low polymorphism rate, and so the likelihood that a single read spans multiple SNPs is small. While matching with up to a few differences (allowing for a SNP and 1–2 errors) is sufficient in these regions, these methods fail when the polymorphism level is high.

NGS technologies are also opening the door to the study of population genomics of non-model individuals in other species. Various organisms have a wide range of polymorphism rates - from 0.1% in humans to 4.5% in the marine ascidian *Ciona savignyi*. The polymorphisms present in a particular species can be used to discern its evolutionary history and understand the selective pressures in various genomic loci. For example, the large amount of variation in *C. savignyi* (two individuals' genomes are as different as Human and Macaque) was found to be due to a large effective population size [11]. The re-sequencing of species like *C. savignyi* (and regions of the human genome with high variability) requires methods for short read mapping that allow for a combination of several SNPs, indels, and sequencing errors within a single (short) read. Furthermore, due to larger-scale “structural” variation, only a fraction of the read may match to the genome, necessitating the use of local, rather than global, alignment methods.

Previous short read mapping tools typically allow for a fixed number of mismatches by separating a read into several sections and requiring some number of these to match perfectly, while others are allowed to vary [4],[6],[8]. An alternative approach generates a set of subsequences from the read (often represented as spaced seeds [7],[10],[12]), again in such a manner that if a read were to match at a particular location with some number of mismatches, at least one of the subsequences would match the genome [5],[9]. While these methods are extremely fast, they were developed for genomes with relatively low levels of polymorphism, and typically cannot handle a highly polymorphic, non-model genome.

This becomes especially apparent when working with data from Applied Biosystem's SOLiD sequencing platform (AB SOLiD). AB SOLiD uses a di-base sequencing chemistry that generates one of four possible calls (colors) for each pair of nucleotides. While a sequencing error is a change of one color-call to another, a single SNP will change two adjacent color positions. Hence a read with two (non-adjacent) SNPs and a sequencing error will differ from the reference genome in five different positions. Simultaneously, the nature of the di-base sequencing code allows for the identification (and correction) of sequencing errors, so by carefully analyzing the exact sequence of matches and mismatches within a read, it is possible to determine that the read and the genome differ by two SNPs. While efficient mappers for color-space sequences have been developed [5],[13], they translate the genome to color-space, and directly compare to the color-space read. The complexity of the color-space representation makes the identification of complex variation such as adjacent SNPs and short indels challenging or impossible with these tools.

In this paper we develop algorithms for the mapping of short reads to highly polymorphic genomes and methods for the analysis of the mappings. We demonstrate an algorithm for mapping short reads in the presence of a large amount of polymorphism. By employing a fast k-mer hashing step and a simple, very efficient implementation of the Smith-Waterman algorithm, our method conducts a full alignment of each read to all areas of the genome that are potentially homologous. Secondly, we introduce a novel, specialized algorithm for mapping di-base (color-space) reads, which allows for an accurate, non-heuristic alignment of AB SOLiD reads to a reference genome. Finally, we introduce methodology for evaluating the accuracy of discovered alignments. Because a read may match the genome in several locations with variable amounts of polymorphism, we develop a statistical method for scoring the hits, allowing for the selection of the most probable variants, and filtering of false positives.

Our methods are implemented as part of SHRiMP: the SHort Read Mapping Package. To demonstrate the usefulness of SHRiMP we re-sequenced a Japanese *Ciona savignyi* genome on the SOLiD platform. Preliminary estimates obtained in the course of sequencing the reference genome indicate that the SNP heterozygosity is 4.5%, whereas indel heterozygosity is 16.6%. This species represents the most challenging known test case for the detection of polymorphisms with short read technologies. We aligned the SOLiD reads of the Japanese individual to the *C. savignyi* reference genome using both SHRiMP and AB's read mapper. SHRiMP is able to identify 5-fold more SNPs than AB's mapper, while also capturing 70,000 indel variants.

## Results/Discussion

**Figure 1:**
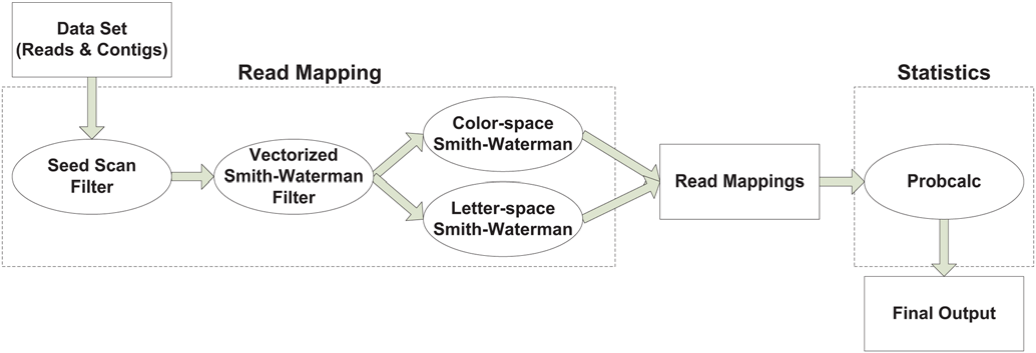
Data flow and processing within the SHRiMP. Candidate mapping locations are first discovered by the seed scanner and then validated by the vectorized Smith-Waterman algorithm, computing only a score. Top scoring hits are then fully aligned by a platform-specific algorithm (i.e. letter-space for Solexa data and color-space for SOLiD data). Statistical confidence for the final mappings are then computed using the PROBCALC utility.

This section is organized as follows: we begin with three methodological sections, in which we first present an overview of the algorithms used in SHRiMP for mapping short reads, explain our specialized algorithm for alignment of di-base sequencing (AB SOLiD) data, and present our framework for computing p-values and other statistics for alignment quality. The data flow for these methods is illustrated in Figure 1. In the last two subsections we will first show the application of SHRiMP to the resequencing of *Ciona savignyi* using the AB SOLiD sequencing technology and present results on the accuracy of the SHRiMP tool on simulated data.

### Read Mapping Algorithm

The SHRiMP algorithm draws upon three recent developments in the field of sequence alignment: q-gram filter approaches, introduced by Rasmussen et al [14]; spaced seeds, introduced by Califano and Rigoutsos [15] and popularized by the PatterHunter family of tools [7],[10]; and specialized vector computing hardware to speed up the Smith-Waterman Algorithm [16]–[18] to rapidly find the likely locations for the reads on the genome. Once these locations are identified, we conduct a thorough, Smith-Waterman-based algorithm to rigorously evaluate the alignments. In this section we will provide a brief exposition of the methods used to align short reads in SHRiMP (a more thorough description of each of these steps is in Methods).

#### Spaced seeds

Most heuristic methods for local alignment rely on the identification of seeds – short exact matches between the two sequences. The advantage of using exact matches is that they are easy to find using hash tables, suffix arrays, or related techniques. While classically seeds have been contiguous matches, more recently “spaced” seeds, where *predetermined* positions in the read are allowed not to match, have been shown to be more sensitive. Spaced seeds are often represented as a string of 1 s and 0 s, where 1 s indicate positions that must match, while 0 s indicate positions that may mismatch. We refer to the *length* or *span* of the seed as the total length of the string, and the *weight* of the seed as the number of 1 s in the string. For example, the seed “11100111” requires matches at positions 1–3 and 6–8, and has length 8 and weight 6. Because seeds with such small weight match extremely often, we require multiple seeds to match within a region before it is further considered, using a technique called Q-gram filtering.

#### Q-gram filters

While most older local alignment tools, such as BLAST, use a single matching seed to start a thorough comparison of the strings around the seed, more recently Rassmussen et al [14] introduced the use of q-gram filters, where multiple seeds are used to determine if a good match exists. This idea is also used in SHRiMP where we require a pre-determined number of seeds from a read to match within a window of the genome before we conduct a thorough comparison.

#### Vectorized Smith-Waterman

If a particular read has the required number of seeds matching to a window of the genome we conduct a rapid alignment of the two regions to verify the similarity. This alignment is done using the classical Smith-Waterman algorithm [19], implemented using specialized “vector” instructions that are part of all modern CPUs. In order to speed up this stage we compute just the score of the optimal alignment, and not the alignment itself. For every read we store the locations of top hits, sorted by their score. The number of top hits to store is a parameter.

#### Final alignment

After we finish aligning all of the reads to all of the potential locations, we conduct a final, full alignment of each read to all of the top hits. This final alignment stage differs depending on the specifics of the sequencing technology. Within SHRiMP we have implemented separate final alignment modules for Illumina/Solexa data (this is done with the regular Smith-Waterman algorithm) and for color-space (di-base) data produced by the AB SOLiD instrument (described in the next section). Additionally we have an experimental module for alignment of two-pass sequencing data, where two reads are generated from every genomic location, which is described elsewhere [20].

### Algorithm for Color-space Alignment

**Figure 2:**
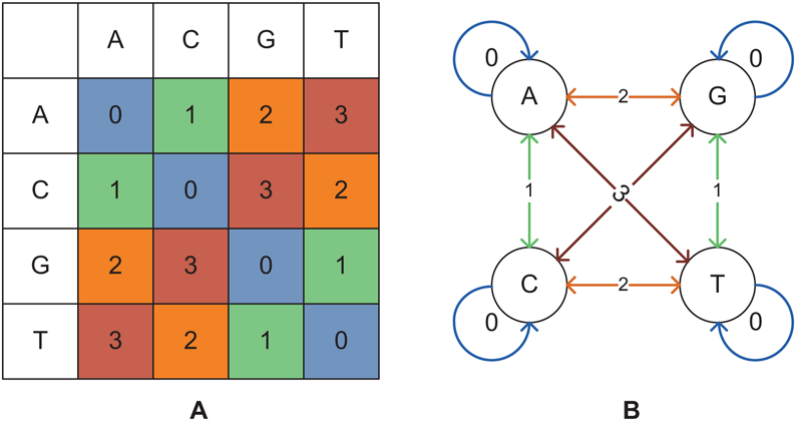
Two representations of the color-space (dibase) encoding used by the AB SOLiD sequencing system. A: The standard representation, with the first and second letter of the queried pair along the horizontal and vertical axes, respectively. B: The equivalent Finite State Automaton representation, with edges labelled with the readouts and nodes corresponding to the basepairs of the underlying genome.

The AB SOLiD sequencing technology introduced a novel dibase sequencing technique, which reads overlapping pairs of letters and generates one of four colors (typically labelled 0–3) at every stage. Each base is interrogated twice: first as the right nucleotide of a pair, and then as the left one. The exact combinations of letters and the colors they generate are shown in Figure 2A. The sequencing code can be thought of as a finite state automaton (FSA), in which each previous letter is a state and each color code is a transition to the next letter state. This automaton is demonstrated in Figure 2B. It is notable that the sequence of colors is insufficient to reconstruct the DNA sequence, as reconstruction requires knowledge of the first letter of the sequence (or the last letter of the primer, which is fixed for a single run of the technology).

The AB SOLiD sequencing technology has the remarkable property of differentiating between sequencing errors and biological SNPs (under the assumption that the reference genome has no sequencing errors): a SNP changes two adjacent readouts of the color-space code, while a sequencing error is unlikely to happen at two adjacent positions by chance (the technology does not sequence adjacent letters at adjacent time points). At the same time, however, the color-space code introduces certain complexities.

**Figure 3:**
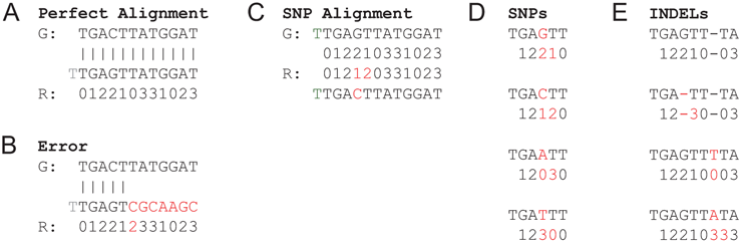
Various mutation and error events, and their effects on the color-code readouts. The reference genome is labeled G and the read R. A: A perfect alignment; B: In case of a sequencing error (the 2 should have been read as a 0) the rest of the read no longer matches the genome in letter-space; C: In case of a SNP two adjacent colors do not match the genome, but all subsequent letters do match. However, D: only 3 of the 9 possible color changes represent valid SNPs; E: the rules for deciding which insertion and deletion events are valid are even more complex, as indels can also change adjacent color readouts.

Let us consider a comparison done by first translating the color-space read code into the letter-space sequence. Notice that a single sequencing error would cause every position after the place of error to be mistranslated (Figure 3B). Consequently, most approaches have translated the letter-space genome into the corresponding color code. However, this is problematic: since the color-coding of every dibase pairing is not unique, a string of colors can represent one of several DNA strings, depending on the preceding base pair. For example, a string of zeroes could be translated as a poly-A, poly-C, poly-G or poly-T string.

There is an additional drawback to translating the genome into color-space code: a sequence of matches and mismatches in color-space does not map uniquely into letter-space similarity. For example, a single SNP results in two sequential color-space mismatches. However, given two consecutive colors, there are 9 possible ways to generate two mismatches. Of these, only 3 correspond to a SNP, while the rest lead to DNA strings that completely differ from the reference. This is illustrated in Figure 3D.

**Figure 4:**
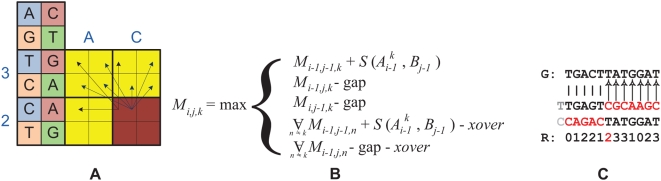
Color-space (dibase) sequence alignment. A: The Dynamic Programming (DP) representation, B: recurrences, and C: alignment of a letter space sequence to a color-space read with a sequencing error. Within the DP matrix we simultaneously align all of the four possible translations (vertical) to the reference genome (horizontal); however the alignment can transition between translations by paying the crossover penalty. This is illustrated by the fourth recurrence, where the third index (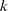) corresponds to the translation currently being used. In the alignment (C) after the sequencing error, the original translation of the read (starting from a T) no longer matches, but a different one (starting from a C) does.

We propose an alternate approach. Our key observation is that while a color-space error causes the rest of the sequence to be mistranslated, the genome will match one of the other three possible translations. This is illustrated in Figure 4C. Consequently, we adapt the classical dynamic programming algorithm to simultaneously align the genome to all four possible translations of the read, allowing the algorithm to move from one translation to another by paying a “crossover”, or sequencing error penalty. If one wishes for a probabilistic interpretation of the algorithm, one can consider the FSA in Figure 2B to be a Hidden Markov Model, where the letter is the hidden state, and the color-space sequence is the output of the model. By taking the cross product of this HMM with the standard pair-HMM associated with the Smith-Waterman algorithm, we can allow all of the typical alignment parameters, including the error penalty, to be probabilistically motivated as the log of the probability of the event, and trained using the Expectation-Maximization algorithm. It is notable that our approach handles not only matches, mismatches, and sequencing errors, but also indels. Because the sequences are aligned in letter-space (to be precise, they are aligned and translated simultaneously), indels can be penalized using the standard affine gap penalty with no further modification of the algorithm.

In the SHRiMP algorithm, we only apply the special color-space Smith-Waterman algorithm in the final stage. For the initial stages, we convert the genome from letter-space to color-space, and search for k-mer matches as well as perform vectorized Smith-Waterman strictly in color-space. In order to better incorporate SNPs in color-space data, we use a spaced seed that allows for two adjacent mismatching colors between the read and the reference genome.

### Computing Statistics for Reads and Mate-pairs

Once all of the reads are mapped, for every read and mate-pair we compute mapping confidence statistics. Initially these are computed for each read; however, they are then combined to compute likelihoods of accidental matches for mate-pairs.

#### Computing statistics for single reads

While a very thorough statistical theory for local alignments has been established [21], this theory assumes the comparison of infinite length strings, and hence is inappropriate for evaluating alignments of very short reads to a reference genome. Instead, we have designed confidence statistics that explicitly model short reads, and allow for the computation of confidences in the presence of short insertions and deletions. We estimate the confidence in the possible mappings of each read by using the following statistics (calculated by the PROBCALC program): *pchance* – the probability that the hit occurred by chance – and *pgenome* – the probability that the hit was generated by the genome, given the observed rates of the various evolutionary and error events. For example, a good alignment would have a low *pchance* (close to 0) and a very high *pgenome* (close to 1). In this section we briefly expand on these two concepts, give them mathematical definitions, and merge them to formulate an overall alignment quality measurement. A detailed description is in Methods (Computing Statistics: *pchance* and *pgenome*).

The *pchance* of a hit is the probability that the read will align with as good a score to a genome that has the same length, but random nucleotide composition with equal base frequencies (that is, the read will align as well *by chance*). In order to compute this, we count all of the possible k-mers with an equal number of changes as observed in the hit, and we call this number 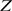. For example, if we only have substitutions in our alignment (that is, no indels) and an alignment length of 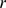, then 
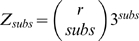
 gives the number of unique strings to which the read can align with the specified number of substitutions. A more detailed discussion on the construction of 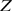, especially for the more complex 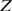 count for indels, appears in Computing Statistics: *pchance* and *pgenome*. The term 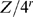 compares the number of unique strings with the given score (when aligned to the read) compared to all possible unique reads of length 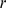, and gives us the probability that a read matches by chance at any location. To compute the *pchance* statistic over the entire length of the genome, we assume independence of positions, and evaluate the likelihood that there is a match at any of the positions:
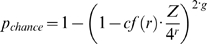
(1)where 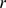 is the alignment length, 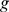 is the genome length (2 corresponds to the two strands), and 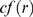 is a correction factor for mappings that are shorter than the length of the read, detailed in Computing Statistics: *pchance* and *pgenome*.

Our second computation, *pgenome*, defines the probability that a hit was generated by the genome via common evolutionary events characteristic of the genome - i.e. substitutions, indels and errors. First, we estimate the rate for each type of event via bootstrapping. Then, we compute the likelihood that the read will differ by as many events from the genome via a binomial probability that uses this estimation and our observations for the events in the current hit. For example, when considering the number of errors, we first estimate the average error rate 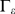 over all hits, and then we can define the probability that the current read was created via this many errors by
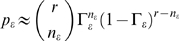
(2)where 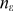 is the number of observed errors in the current hit, and 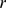 is the alignment length. We can similarly define 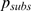 and 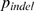 for substituion and indel events, respectively. Finally, we can form 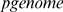 as
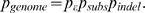
(3)More specifics about the mathematical formulations are available in Computing Statistics: *pchance* and *pgenome*.

Finally, we define the quality measurement of this hit as the *normalized odds*, i.e. a probability odds ratio 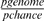 normalized over all of the hits of this read:
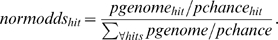
(4)This value represents a relative credibility of this hit compared to the others for a given read: A single hit would have a normalized odds score of 1, two equally good hits will both have 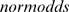 of 0.5 for both, while for an exact match and a more distant one, the former will have a 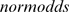 close to 1, and the latter close to 0.

#### Computing statistics for mate-pairs

SHRiMP also assigns mate-pair confidence values (akin to the read confidence values predicted by probcalc) by combining the confidence values for individual reads with emprically observed distributions of insert sizes in the library. We compute the distribution of the mapped distances (distance between the mapped positions of the two reads) 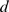 for all mate-pairs, and save the average distance 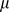 (see *Computing Mate Pairs with Statistics* for more details). Then, for each mate-pair mapping, we assign a pchance, pgenome and normodds score, similar in meaning to those used in the previous section:

- ***pchance*** **for mate-pairs**: assume 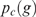 is the pchance of a read that takes g, the length of the genome, as a parameter. Now, the pchance of a mate-pair read_1, read_2 is defined as
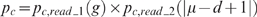
(5)where 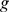 is the length of the genome used in probcalc, 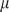 is the average mate-pair distance, and 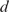 is the distance of the current mate-pair. That is, we ask the question: what is the probability that a read as good as the first read would align anywhere in the genome by chance, *and* that a second read will align by chance within the observed mate-pair distance?
- ***pgenome*** **for mate-pairs**: assume 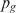 is the pgenome of a read. We can compute the pgenome of each mate-pair by
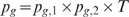
(6)where 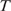 is the tail probability of the mate-pair distance distribution we computed (both tails, starting at the 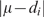 cutoff). Therefore, for a mate-pair with the distance really close to 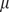, the pgenome will be close to 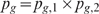, otherwise, it will be penalized. Thus, following the difinition of pgenome, we will get a lower probability that the mate-pair was generated from the genome if the mate-pair distance is too big or too small compared to the average.

A discussion of the implementation steps are included in the SHRiMP README, and a more detailed discussion of the statistical values is included in *Computing Mate Pairs with Statistics*.

### Validation

In our experiments, we used SHRiMP to compare 135 million 35 bp reads from a tunicate *Ciona savignyi* to the reference genome [22]. The fragments were sequenced from sheared genomic DNA with an AB SOLiD 1.0 instrument. In the following sections we first describe the running time of SHRiMP at different parameter settings, and then evaluate the quality of our alignments compared to the Applied Biosystem's read mapping program.

#### Running time analysis

One of the advantages of the SHRiMP algorithm is the seamless parallelism provided by the fact that we can simply subdivide the reads into separate computational jobs, without affecting the results. This allows us to take full advantage of compute clusters regardless of the amount of memory available at each machine. We took a random subset consisting of 500,000 35 bp *C. Savignyi* reads and mapped them to the genome. The full read dataset and reference genome are available at http://compbio.cs.toronto.edu/shrimp/misc/paper_ciona_reads_35mer.csfasta.tar.bz2 and http://mendel.stanford.edu/sidowlab/CionaData/CionaSavignyi_v.2.1.fa.zip, respectively.

The running times at several parameter settings are summarized in Table 1. Note that from smallest to largest seed weight, we see a nearly two orders of magnitude difference in total run time, most of which is concentrated in the vectorized Smith-Waterman filter, and, to a lesser degree, in the spaced k-mer scan. The final, full color-space Smith-Waterman alignment took approximately constant time across all runs, as the average number of top scoring hits that reached the stage was nearly constant (24.49±0.5); however, proportional time increased as the filter stages became more efficient. While SHRiMP is somewhat slower than other short read mapping programs, it allows both for micro-indels in the alignments and a proper color-space alignment algorithm. SHRiMP is also very configurable in terms of sensitivity and running time trade-offs.

**Table 1:** Running time of SHRiMP for mapping 500,000 35 bp SOLiD *C. savignyi* reads to the 180 Mb reference genome on a single Core2 2.66 GHz processor.

K-mer	(7,8)	(8,9)	(9,10)	(10,11)	(11,12)	(12,13)
% K-mer Scan	10.1%	16.5%	18.9%	13.4%	9.8%	7.4%
% Vectorized SW Filter	88.8%	75.4%	49.8%	30.2%	20.1%	14.9%
% Full SW Alignment	1.1%	8.0%	30.7%	55.5%	68.8%	76.2%
Time	1 d21 h34 m	6 h18 m	1 h36 m	50 m28 s	37 m52 s	32 m32 s

In all cases, two k-mer hits were required within a 41 bp window to invoke the vectorized Smith-Waterman filter.

#### *Ciona savignyi* polymorphism analysis

The primary strength of SHRiMP and other mapping methods based on Smith-Waterman alignments is the ability to map reads containing complex patterns of sequence variation, including insertions, deletions and clusters of closely-space SNPs. Mappers that exclusively produce ungapped alignments can only find SNPs. Furthermore they are more likely to miss dense clusters of SNPs, since the overlapping reads contain many mismatches, and SNPs adjacent to an indel, since only a small fraction of the overlapping reads contain just the SNP. Finally, since SHRiMP produces local alignments, it can map a read even if either end overlaps a large indel or structural variant.

To evaluate the effectiveness of SHRiMP for detecting sequence variation we used it to find polymorphisms in a resequenced *Ciona savignyi* individual. *C. savignyi* is a challenging test case because of its very high polymorphism rate: the SNP heterozygosity is 4.5% and the average per-base indel heterozygosity is 16.6% (indel rate of 0.0072 events per base) [11]. We therefore expect that even short reads will frequently span multiple variant sites.

We used the AB SOLiD sequencing platform to generate 135 million reads of length 35 bp from a single *C. savignyi* individual. We then aligned those reads to the reference genome [22] with SHRiMP using lenient scoring thresholds so that reads with multiple variant sites could be mapped, and we selected the single highest-scoring alignment for each read (see Methods). We discarded alignments in repetitive sequence by removing reads with multiple similarly scoring alignments (“non-unique” matches). The mapping took 48 hours using 250 2.33 GHz cores. Table 2 summarizes the mapping results.

**Table 2:** Mapping results for 135 million 35 bp SOLiD reads from *Ciona savignyi* using SHRiMP and the SOLiD mapper provided by Applied Biosystems.

	SHRiMP	SOLiD Mapper
Uniquely-Mapped Reads	51,856,904 (38.5%)	15,268,771 (11.3%)
Non-Uniquely-Mapped Reads	64,252,692 (47.7%)	12,602,387 (9.4%)
Unmapped Reads	18,657,736 (13.8%)	106,896,174 (79.3%)
Average Coverage (Uniquely-Mapped Reads)	10.3	3.0
Median Coverage (Uniquely-Mapped Reads)	8	1
SNPs	2,119,720	383,099
Deletions (1–5 bp)	51,592	0
Insertions (1–5 bp)	19,970	0

Non-uniquely-mapped reads have at least two alignments, none of which is significantly better than the others (see Methods). SNPs and indels have at least four supporting reads.

The alignment data contains noise due to two types of errors: sequencing errors and chance alignments. Chance alignments are a significant problem for short reads, particularly with the low alignment score thresholds necessary for mapping reads containing significant variation. Reads containing both sequence variation and sequencing errors are even more likely to map to the wrong position in the reference sequence. To combat the high false-positive rate, for the remaining analysis we focused on a high-quality subset of the data consisting of sequence variants supported by at least four independent reads.

Across the genome SHRiMP detected 2,119,720 SNPs supported by at least four reads. For comparison, we used the SOLiD aligner provided by Applied Biosystems to map the reads to the genome with up to three mismatches, where each mismatch can be either a single color-space mismatch or a pair of adjacent mismatches consistent with the presence of a SNP. Compared to the SOLiD mapper, SHRiMP mapped 4.2 times as many reads and found 5.5 times as many SNPs. The AB mapper, however, was a lot faster, requiring 255 CPU hours to complete the alignments, or roughly 50× faster than SHRiMP. While it is possible to run the mapper with greater sensitivity, allowing for more errors and SNPs, and thus more mapped reads, doing so would surrender much of the runtime advantage and still not overcome its fundamental inability to detect insertion and deletion polymorphisms. SHRiMP, on the other hand, is capable of handling indels, and detected tens of thousands of them.

**Figure 5:**
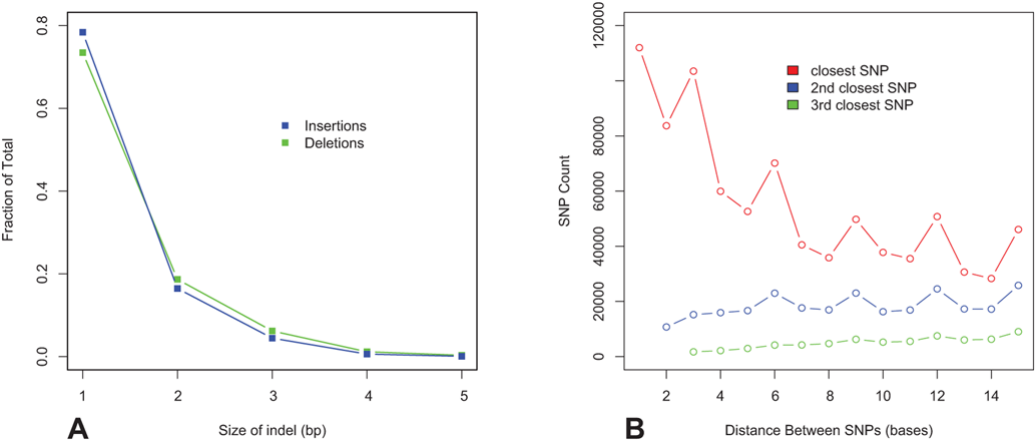
Size distribution of indels. (A) and distance between adjacent SNPs (B) detected by SHRiMP. The distance between adjacent SNPs shows a clear 3-periodicity, due to the fact that a significant fraction of the non-repetitive *C. savignyi* genome is coding.

SHRiMP detected 51,592 deletions and 19,970 insertions of size 1–5 bp. The observed ratio of 2.5× between insertions and deletions for the *C. savignyi* data is biased by the construction of the reference genome – whenever the two haplomes differed, the reference agreed with the longer one. While there is a smaller inherent bias against detecting insertions (reads containing nucleotides not present in the reference) compared to deletions because a read spanning a deletion only incurs a gap penalty whereas an insertion both incurs a gap penalty and has fewer bases that match the reference. For simulated data (see next section) this bias was only ∼5% for single basepair indels (data not shown). The size distribution of the detected indels (Figure 5A) drops more rapidly with length than expected [11], but this detection bias against longer indels is not surprising since longer indels have lower alignments scores.

Mapping *C. savignyi* sequence is challenging primarily because the population contains so much variation. Figure 5B shows the high frequency of closely spaced SNPs detected by SHRiMP. Mappers that can only detect nearly exact matches fail to map the reads overlapping these dense SNP clusters. Note that even though the reads are generated from the whole genome, a significant fraction of the non-repetitive *C. savignyi* genome is coding, making it is possible to see the typical three-periodicity of SNPs in coding regions. Furthermore SHRiMP recovers microindels, which are completely invisible to ungapped aligners and yet account for a significant fraction of sequence variation in *C. savignyi*.

#### Analysis of simulated data

In order to further validate the accuracy of the SHRiMP alignments we have designed simulated experiments, where we sampled random locations from the *C. savignyi* genome, introduced polymorphisms (SNPs and indels) at the rates previously observed in the C. savignyi genome [22], added sequencing errors at rates observed in our *C. savignyi* dataset (2–7%, depending on the position in the read), and mapped the reads back to the original genome. Each sampled read could have multiple SNPs and indels, though due to the low indel rate only a small fraction of the reads had multiple indels. We mapped the reads with SHRiMP and postprocessed with PROBCALC (*pchance*<0.001). Considering only those reads that had a unique top hit, we computed the **precision** – the fraction of reads for which this unique hit was correct, and **recall** – the fraction of all reads that had a unique, correct hit. Table 3 shows the results of this analysis. For each read, we classified it based on the number of SNPs and the maximum indel length, and computed precision and recall for each class. With such polymorphism, we can expect the average read to have approximately 1.5 SNPs and 1.9 errors. SHRiMP was able to accurately map 76% of reads with 2 SNPs and 0 indels, at 84% precision, and nearly half of all reads with 2 SNPs and 3 bp indels at 74% precision.

**Table 3:** Color-space mapping accuracy of SHRiMP.

		Number of SNPs									
		0		1		2		3		4	
		Prec.	Rec.	Prec.	Rec.	Prec.	Rec.	Prec.	Rec.	Prec.	Rec.
	0	85.7	83.2	84.8	81.3	83.5	76.6	80.6	65.2	75.6	46.8
Max	1	83.8	79.4	82.2	74.0	79.4	62.6	72.8	43.2	63.1	24.7
Indel	2	83.2	77.1	80.8	69.6	77.9	56.6	68.2	36.4	56.4	18.9
Length	3	80.7	71.0	79.6	64.2	73.6	48.3	66.5	31.5	57.1	16.6
	4	78.0	65.4	76.5	56.1	71.4	41.9	60.6	23.9	50.3	12.4
	5	75.9	58.9	73.0	48.1	69.7	36.6	57.0	21.3	46.0	12.7

Each cell shows the precision and recall for mapping simulated reads with varying amounts of polymorphism. SHRiMP was able to accurately map >46% of all reads with either 4 SNPs or 5 bp indels, despite the large number of sequencing errors in our dataset (up to 7% towards the end of the read).

## Methods

### Details of the SHRiMP Algorithm

**Figure 6:**
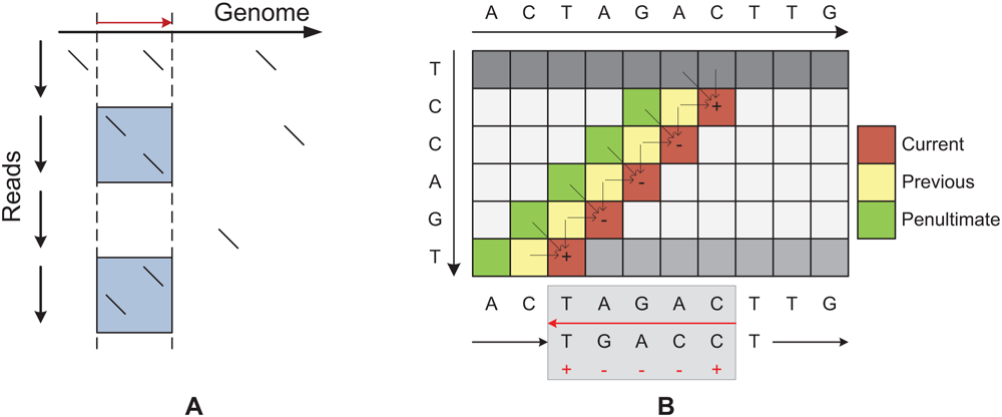
SHRiMP Hashing technique & Vectorized Alignment algorithm. A: Overview of the k-mer filtering stage within SHRiMP: A window is moved along the genome. If a particular read has a preset number of k-mers within the window the vectorized Smith-Waterman stage is run to align the read to the genome. B: Schematic of the vectorized-implementation of the Needleman-Wunsch algorithm. The red cells are the vector being computed, on the basis of the vectors computed in the last step (yellow) and the next-to-last (blue). The match/mismatch vector for the diagonal is determined by comparing one sequence with the other one reversed (indicated by the red arrow below). To obtain the set of match/mismatch positions for the next diagonal, the lower sequence needs to be shifted to the right.

The algorithm starts with a rapid k-mer hashing step to localize potential areas of similarity between the reads and the genome. All of the spaced k-mers present in the reads are indexed. Then for each k-mer in the genome, all of the matches of that particular k-mer among the reads are found. If a particular read has as many or more than a specified number of k-mer matches within a given window of the genome, we execute a vectorized Smith-Waterman step, described in the next section, to score and validate the similarity. The top 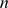 highest-scoring regions are retained, filtered through a full backtracking Smith-Waterman algorithm, and output at the end of the program if their final scores meet a specified threshold. The SHRiMP algorithm is summarized in Figure 6.

#### Spaced seed filter

We build an index of all spaced k-mers in the reads, and query this index with the genome. Our approach was taken primarily for simplicity: our algorithm can rapidly isolate which reads have several k-mer matches within a small window by maintaining a simple circular buffer of recent positions in the genome that matched the read. Since our targeted compute platform is a cluster of batch processing machines, indexing the reads means that we can easily control memory usage and parallelism by varying the read input size and splitting the read set accordingly. Data is only loaded at program invocation; we do not stream in new reads from disk as the algorithm runs.

#### Vectorized Smith-Waterman implementation

The SHRiMP approach relies on a rather liberal initial filtering step, followed by a rigorous, but very fast Smith-Waterman alignment process. By maximizing the speed of the Smith-Waterman comparison, we are permitted to let the algorithm test a larger number of potential regions.

Most contemporary mobile, desktop and server-class processors have special *vector* execution units, which perform multiple simultaneous data operations in a single instruction. For example, it is possible to add the eight individual, 16-bit elements of two 128-bit vectors in one machine instruction. Over the past decade, several methods have been devised to significantly enhance the execution speed of Smith-Waterman-type algorithms by parallelizing the computation of several cells of the dynamic programming matrix. The simplest such implementation computes the dynamic programming matrix using diagonals. Since each cell of the matrix can be computed once the cell immediately above, immediately to the left, and at the upper-left corner have been computed, one can compute each successive diagonal once the two prior diagonals have been completed. In this way, the problem can be parallelized across the length of supported diagonals (see Figure 6B). In most cases, this is a factor of 4 to 16. The only portion of such a ‘Wozniak’ approach that cannot be parallelized is the identification of match/mismatch scores for every cell of the matrix, which has to be done sequentially. These operations are expensive, necessitating 24 independent data loads for 8-cell vectors, and become increasingly problematic as vector sizes increase. Because memory loads cannot be ‘vectorized’, when the parallelism grows, so does the number of lookups. For example, with 16-cell vectors, the number of data loads doubles to 48.

We propose an alternate method, where the running time of the fully vectorized algorithm is independent of the number of matches and mismatches in the matrix, though it only supports fixed match/mismatch scores (rather than full scoring matrices). Our key observation is that it is possible to completely parallelize the score computation for every diagonal. Figure 6B demonstrates the essence of our algorithm: by storing one of the sequences backwards, we can align them in such a way that a small number of logical instructions obtain the positions of matches and mismatches for a given diagonal. We then construct a vector of match and mismatch scores for every cell of the diagonal without having to use expensive and un-vectorizable load instructions or a pre-compute a ‘query profile’. In our tests, using a diagonal approach with our scoring scheme surpasses the performance of Wozniak's original algorithm and performs on par with Farrar's method [17]. Table 4 summarizes these results. The advantage of our method over Farrar's is that it is independent of the scores used for matches/mismatches/gaps, and it will scale better with larger vector sizes. A disadvantage is that we cannot support full scoring matrices and are restricted to match/mismatch scores, though this is less important for DNA alignment. Additionally, Farrar's method is much faster for large databases where most of the sequence is dissimilar to the query. However, this is never the case for SHRiMP as the seed scan phase targets only small, similar regions for dynamic programming. In these cases our algorithms perform similarly.

**Table 4:** Performance (in millions of cells per second) of the various Smith-Waterman implementations, including a regular implementation (not vectorized), Wozniak's diagonal implementation with memory lookups, Farrar's method and our diagonal approach without score lookups.

Processor type	Unvectorized	Wozniak	Farrar	SHRiMP
**Xeon**	97	261	335	338
**Core 2**	105	285	533	537

We inserted each into SHRiMP, and used SHRiMP to align 50 thousand reads to a reference genome with default parameters. The improvements of the Core 2 architecture for vectored instructions lead to a significant speedup for our approach and Farrar's, while Wozniak's algorithm slight improvement is due to the slow match/mismatch lookups.

#### Final pass

The vectorized Smith-Waterman approach described above is used to rapidly determine if the read has a strong match to the local genomic sequence. The locations of the top 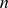 hits for each read are stored in a heap data structure, which is updated after every invocation of the vectorized Smith-Waterman algorithm if the heap is not full, or if the attained score is greater than or equal to the lowest scoring top hit. Once the whole genome is processed, highest scoring 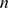 matches are re-aligned using the appropriate full color- or letter-space Smith-Waterman algorithm. This is necessary, as the vectorized Smith-Waterman algorithm described above only computes the maximum score of an alignment, not the traceback, as this would require a much more complicated and costly implementation. Instead, at most only the top 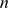 alignments for each read are re-aligned in the final step.

### Computing Statistics: *pchance* and *pgenome*

In *Computing Statistics for Single Reads*, we briefly introduced the concepts of the *pchance*, *pgenome* and *normalized odds* of a hit. In this section we expand on the details regarding the construction of *pchance* and *pgenome*. In these formulas we make use the following definitions:

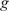
 is the genome length

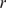
 is the alignment length (note this may be different from the read length, which is constant)

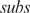
 is the number of substitutions (mismatches) in our alignment

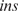
 is the number of nucleotide insertions in our alignment, where the genome is the “original” sequence. For example, if the genome is AC-G and a read is ACTG, there is an insertion of a T.

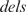
 is the number of nucleotide deletions in our alignment. For example, if the genome is ACTG and a read is A-TG, there is a deletion of a C.

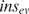
 is the number of insertion events (for example, for a single insertion of length 3 we have 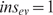 and 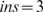.) 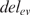 is similar.

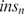
: following the previous definition, 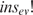 will describe the number of permutations of insertion events. To determine the number of *distinguishable* permutations, we need to first look at the frequency of insertion events of a certain size, 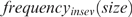. For example, is we have 3 insertions of size 2, we need to divide the permutations by 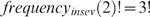. Therefore, the *distinguishable* permutations of insertion events can be written as:
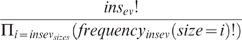

Below, we refer to this denominator term 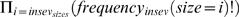 as 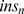. We similarly define 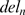.

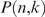
 describes the number of ways to assign 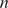 indistinguishable objects in 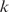 indistinguishable bins, which is recursively defined by 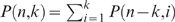 with 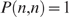 and 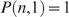.

#### pchance

We begin with the mathematical formulation of *pchance* (defined above):
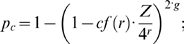
(7)where, as described before, 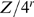 is the number of possible unique sequences with the given edit distance as a fraction of all possible unique reads of length 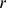. Thus, 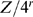 gives us the probability that the current read has aligned by chance to a random genome of the size of a read. To this term, we add a correction factor of 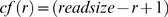 which accounts for all the possible places the alignment of size 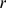 might match. For example, if the 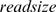 is 25 and we have a match of size 22, we should count 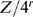 for every position where this match could be found, that is 25−22+1 = 4. Finally, to get the probability that the current read has aligned by chance to a random genome of size 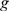 (instead of size 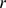), we get formula (7).

The factor that lies at the core of this calculation is 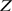, the number of possible unique sequences that would align to the read with the given edit distance. We have shown the definition of 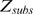, which computes 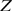 when there are no indels in the alignment:
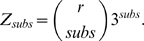
(8)


However, the calculation of the number of references to which a read will map with a particular *indel* count, 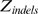, depends on the sequence of that read and is significantly more complicated. We define a lower and upper bound on 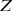 in this case: a lower bound (least number of unique sequences) occurs when the current read is one repeated nucleotide, for example [AAAAAA], and the higher bound occurs with the most change in nearby nucleotides, say [ACGTAC]. In the former case, we need to look at the deletion events from the genome to this read, consider all the combinations of that number of deletion events and deleted nucleotides, as well as all the places where these combinations may occur. This gives the formula
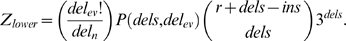
(9)Looking for the upper bound, we note that the places and combinations of insertions also matters in generating unique sequences, therefore giving us two extra terms involving 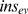


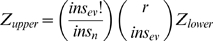
(10)

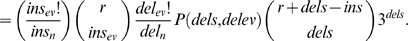
(11)


In order to estimate the correct value for 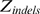, we estimated the average complexity of the reads in our dataset (i.e., between the simplest [AAAAA…] and the most complex [ACGTACGT…]). And have found that the mean observed 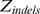 could be accurately estimated by
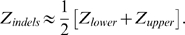
(12)Finally, we can approximate the total 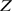 as
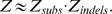
(13)


#### pgenome

In *Computing Statistics for Single Reads*, we defined our pgenome factor as 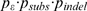, where
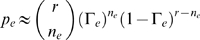
(14)with 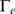 the rate of event 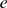 (estimated via bootstrapping) and 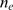 the number of observed events of type 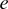 in the current alignment. We wrote 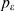 as an approximation because there are small corrections to this formula for each probability that is part of pgenome. First, for the *error* term 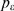, the number of sites that can support errors is in fact one minus the read size, giving us
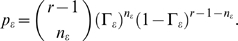
(15)When considering substitutions, we can have changes at any of the inner nucleotides, excluding erroneous sites:
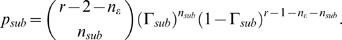
(16)As before, when we look at alignments that involve indels, the formula becomes more complex. In the case of *pgenome*, we do not have to consider the various placements of insertion or deletion events, but we do have to consider, for fixed placements of events, the various combinations of the total number of insertions and deletions into a set number of events.
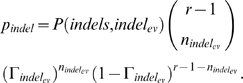
(17)


#### Computing mate pairs with statistics

In this section we provide several details for the implementation, usage and statistics of the matepair post-processing step introduced in *Computing Statistics for Mate-pairs*. We define a **good** matepair mapping as a mapping whose distance 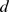 (between the two reads) are smaller than some chosen limit 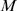, and for which the read mappings are in a consistent orientation and strand(i.e. R_+_F_+_ or F_−_R_−_). First, probcalc_mp will compute a matepair distance and standard deviation by looking at all the connected forward and reverse reads - all matepairs - and adding the distance of any matepair with exactly one *good* mapping to a histogram. Optionally, one can choose to use only unique good mappings, or only use a certain number of mappings (say, the first 100,000) to speed up the program.

Next, we call a matepair **concordant** if it has at least one *good* mapping, and otherwise we call it **discordant**. Depending on the task, probcalc_mp can output all concordant matepairs, or all discordant matepairs. For each matepair mapping, probcalc_mp will compute the pgenome and pchance, as introduced in *Computing Mate Pairs with Statistics*.

### Parameters

For the *C. savignyi* polymorphism analysis we ran SHRiMP with the following parameters. We used the spaced seed “11110111” and required two hits per 40-base window to invoke the Smith-Waterman algorithm. The Smith-Waterman scoring parameters were set to +100 for a matching base, −90 for a mismatch, −250 and −100 to open and extend a gap respectively, and −300 for a crossover (sequencing error). The minimum Smith-Waterman score thresholds were 1000 for the vectorized first pass and 1275 for the final alignment pass. We discarded alignments with *pchance* less than 0.05, and to remove reads from known repetitive sequence we required *normodds* to be at least 0.8.
